# Novel insights into *SOS1* in managing vacuolar sodium toxicity

**DOI:** 10.3389/fpls.2026.1780410

**Published:** 2026-03-20

**Authors:** Md. Mahadi Hasan, Nadiyah M. Alabdallah, Sumayah I. Alsanie, Ghadah Hamad Al Hawas, Md Atikur Rahman

**Affiliations:** 1 Basic and Applied Scientific Research Center, Imam Abdulrahman Bin Faisal University, Dammam, Saudi Arabia; 2 Department of Biology, College of Science, Imam Abdulrahman Bin Faisal University, Dammam, Saudi Arabia; 3 Institute of Biological Sciences, University of Rajshahi, Rajshahi, Bangladesh

**Keywords:** CryoNanoSIMS, root meristematic cells, salinity, salt overly sensitive 1 (*SOS1*) gene, vacuolar Na^+^

## 1 Introduction

Salinization due to the excessive addition of water-soluble salt to soils is a global agricultural concern, with appropriately 20%–40% crop losses due to soil salinity (Tarolli et al., 2024). The majority of agricultural plants are sensitive to toxic sodium (Na^+^) in soils, which impairs plant growth, development, and productivity, resulting in agricultural yield losses of approximately 20%–40% (Ramakrishna et al., 2025). The classical approach to plant salinity tolerance is emphasized in the role of the plasma membrane (PM)-localized sodium (Na^+^)/proton (H^+^) antiporter SALT OVERLY SENSITIVE 1 (*SOS1*) in extruding Na^+^ from the cytoplasm to the outside of cell wall (Xie et al., 2022). This model, which was established in the last two decades, has improved our understanding of plant toxic Na^+^ homeostasis. However, a novel insight into the dual function of *SOS1* in vacuolar Na^+^ sequestration has been explored using cryo-nanoscale secondary ion mass spectrometry (CryoNanoSIMS), although the role of *SOS1* in Na^+^ extrusion is well recognized in plants.

The introduction of the CryoNanoSIMS approach has examined a new dimension of research on the subcellular distribution of ions in their original and hydrated state. This mass spectrometry tool provides a direct opportunity to visualize Na^+^, K^+^, and other key ions at subcellular organelle levels. The application of CryoNanoSIMS in salt stress response explores a deeper and more dynamic picture of how root meristematic cells (RMCs) drive root growth and manage Na^+^ toxicity in response to different salt levels. This opinion manuscript addresses the paradigm-shifting insights, specifically the dual role of *SOS1* in PM Na^+^ export and vacuolar Na^+^ sequestration. I argue on this novel insight and its fundamental necessity for redefining the Na^+^ management strategies that open new opportunities for the design of salinity-smart future crop production. In addition, We propose designing salinity-smart crops with a paradigm shift in the Na^+^ management approaches addressing various issues from apoplastic retention at low salt levels to vacuolar sequestration at high salt levels. It is hoped that these Na^+^ management strategies represent an unprecedented adaptive mechanism of salinity tolerance in different plant species.

## 2 CryoNanoSIMS: a key tool for the detection of multiple subcellular elements

Na^+^ tracking is essential for exploring how plants manage toxic levels of Na^+^ in plant cells. Ramakrishna et al. (2025) explored a CryoNanoSIMS technique by which specimens containing Na^+^ and other elements were observed with a focus beam. The location of Na^+^ in the cytoplasm and its accumulation inside the vacuole were identified in *Arabidopsis* root meristem cells using the CryoNanoSIMS approach. Furthermore, the location of *SOS1* in the tonoplast and its function in the transport of Na^+^ were confirmed in wild-type *Arabidopsis* after considering a comparative study in *sos1–1* mutant plants. However, the study on *Arabidopsis* was extended to rice, which showed almost the same fashion of elemental accumulation inside the vacuole, while the rice *SOS1* transported Na^+^ into the vacuole. These consecutive findings suggest that the CryoNanoSIMS tool is suitable for the model plant *Arabidopsis* and other grain crops. This novel approach with gold standard of elemental imaging provides several key advantages, including identification of the benefits of multiple target ions (e.g., Na^+^, K^+^, and Ca^2+^) at a time. This cryogenic approach maintains the ion distribution at an almost natural state, which is required for avoiding ion redistribution. This tool allows chemical fixation of the sample materials, provides stability that is crucial for the accuracy of the results, and avoids diffusible ion (Na^+^) leaching of cells and tissues. However, CryoNanoSIMS is not a suitable method for elements with atomic mass higher than that of K. Without this aspect, the CryoNanoSIMS method is suitable for Na^+^ tracking, supporting the involvement of *SOS1* in Na^+^ sequestration inside the vacuole. The CryoNanoSIMS-based imaging method adds a new dimension to our understanding of element distribution, homeostasis, and cellular adaptation under salt stress.

## 3 Vacuolar Na^+^ management strategies

Plant roots represent the first sensing organ. In particular, the root meristematic tissue plays a pivotal role in salt sensing, signaling, transport, and storage of Na^+^ ions in response to salt stress (Rahman et al., 2022; Wang et al., 2022). Ramakrishna et al. (2025) recently explored the process of shifting Na^+^ from the cytoplasm to the vacuole in *Arabidopsis* roots. The plant vacuole maintains cell rigidity and stores toxic Na^+^ and other elements that help protect the enzymes present in the cytoplasm. It has been suggested that the Na^+^ accumulation and localization patterns are different from those of K^+^ and other elements in the subcellular organelles. Thus, the tracking of toxic Na^+^ inside plant cells could determine its accumulation patterns into these cells, indicating the hallmark of Na^+^ management processes that help plants deal with salt stress.

**Figure 1:**
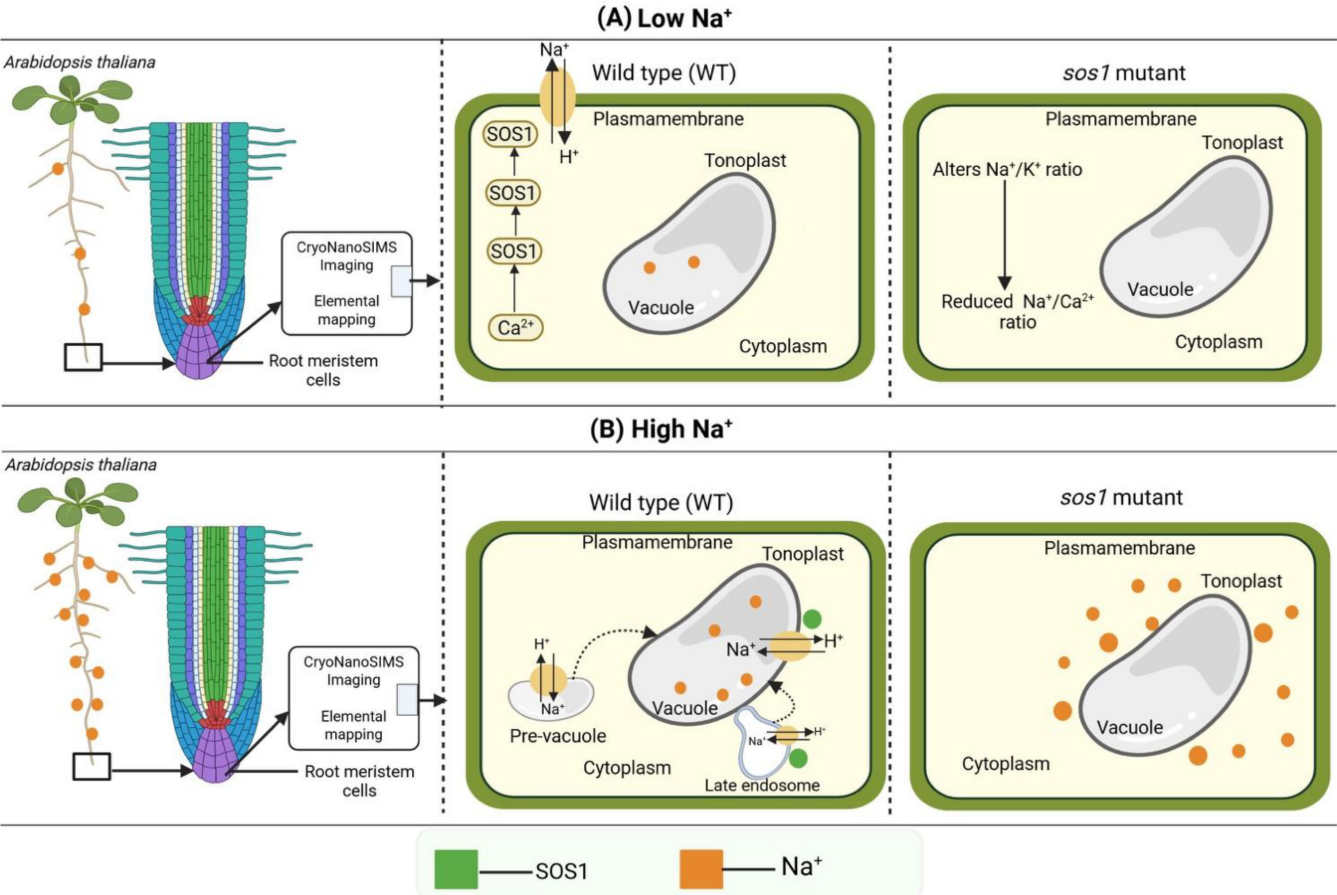
A conceptual model of the mechanisms of vacuolar Na^+^ management strategies in *Arabidopsis* root meristem cells. This figure illustrates the cellular response to low and high Na^+^ comparing wild-type (WT) and mutant plants. **(A)** A low Na^+^ level triggers the Salt Overly Sensitive (SOS) signaling pathway, wherein *SOS3* acts as a Ca^2+^ sensor and *SOS2* a protein kinase, which are known to regulate *SOS1* function in WT plants, while *sos1* alters the Na^+^/K^+^ ratio that leads to the reduction of the Na^+^/Ca^2+^ ratio in the *sos1* mutant. **(B)** At a high Na^+^ level, the tonoplast-localized S*OS1* sequestrates Na^+^ into the vacuole as a vacuolar Na^+^ management strategy, the localization of *SOS1* is discovered at the late endosome or pre-vacuole, and Na^+^ is sequestrated in the late endosome or pre-vacuolar, further delivering to the vacuole through vascular fusion. In the *sos1* mutant lacking a functional *SOS1*, the accumulation of Na^+^ increases in the cytoplasm, displacing cytoplasmic K^+^ and disrupting ionic homeostasis. *Broken arrow* indicates tentative shifting or delivery of Na^+^ into the vacuole. *Na^+^*, sodium ion; *CryoNanoSIMS*, cryogenic nanoscale secondary ion mass spectrometry.

There are several imaging techniques for the investigation of imaging elements and their distribution patterns into plant cells, including particle-induced X-ray emission (microPIXE) and low-energy synchrotron-based X-ray fluorescence microscopy (XFM). The limitation of the fluorescent sensor or radioisotope-based elemental imaging approach is that it can identify only a single target ion at a time. CryoNanoSIMS is a concentration-dependent approach for localizing Na^+^ patterns. For instance, at low external Na^+^ levels (≤2.5 mM salt), the Na^+^ is mainly accumulated in the cell walls and there is low presence of Na^+^ in the cytoplasm. However, in moderate to high saline conditions (25–100 mM salt), wild-type plants showed a significant redirection of the Na^+^ into the vacuole, while the *sos1* mutant exhibited a reverse pattern that enhanced the Na^+^ in the cytoplasm and declined vacuolar accumulation (**Figure 1**). These findings suggest that plants possess distinct detoxification mechanisms dependent on the intensity of salt stress.

At low Na^+^ levels, controlling Na^+^ in the apoplast through minimizing accumulation or moderate Na^+^ extrusion is metabolically possible. However, as the external Na^+^ increases to significant toxic levels, this strategy becomes inefficient. In this scenario, cells require better active salt tolerance mechanisms such as vacuolar sequestration that separates the Na^+^ from cytoplasmic processes during cellular osmotic balance. The molecular mechanism that triggers this shifting, specific function of increasing the cytosolic Na^+^ concentration and the sensing mechanism that recognizes apoplastic Na^+^ levels are still unclear. A recent study has recognized that *SOS1* primarily regulates Na+ efflux at the plasma membrane rather than simply altering total cellular Na^+^ accumulation (Ramakrishna et al., 2025). It has already been recognized that *SOS3* acts as a Ca^2+^ sensor and *SOS2* as a protein kinase, which, together, are known to regulate *SOS1* activity. However, the function of *SOS1* in the PM *versus* the endomembrane (EM) is still a fascinating area of exploration. The recent discovery of *SOS1* localization in the late endosome (LE) and perivacuolar compartments, along with the PM and tonoplast, has provided a mechanistic insight for advancing our understanding of vacuolar Na^+^ accumulation (Ramakrishna et al., 2025). This particular mechanistic framework is fascinating as it suggests that Na^+^ is sequestrated into the endosomal compartment (EC) and the way to the vacuole, except it is transported directly across the tonoplast (**Figure 1**). Thus, the intensity of the surface-to-volume ratio of the vacuolar compartment (VC) provides a new aspect of understanding rapid Na^+^ uptake and management strategies for toxic Na^+^ from the vacuole.

## 4 *SOS1* is involved in new roles

*SOS1* performs a dual function: Na^+^ extrusion and vacuolar sequestration or compartmentation. The mechanism of Na^+^ extrusion has already been explored. Recently, Ramakrishna et al. discovered novel insights into the role of *SOS1* in the rapid sequestration of Na^+^ into the EC followed by the vacuoles (**Figure 1**). In this mechanism, the first line of defense comprises restricting the Na^+^ uptake. The second tier involves triggering the PM *SOS1*-based extrusion of the Na^+^ that is active and efficient at a low concentration (**Figure 1A**). In contrast, a high level of Na^+^ enhances stress, and the extrusion mechanisms are not able to manage Na^+^ toxicity. In the third tier, the endosome-localized *SOS1* sequestrates Na^+^ into the endosomes, followed by its delivery to the vacuole through vascular fusion (**Figure 1B**). In the fourth tier, the tonoplast-localized *SOS1* is involved in the tonoplast-based requisition of Na^+^, a process that helps manage toxic Na^+^ levels. The differential observations raised several questions, including why the *sos1* mutant shows high defects in response to high salt stress, but not in low, possibly indicating that the early tiers are partially active at low salt levels. Moreover, the reason for vacuolar *NHX* knockout mutants exhibiting a moderate phenotype could be that *SOS1* might be compensated (Bassil et al., 2019). The above processes suggest that coordination is crucial across all links for optimum salt tolerance in plants.

## 5 Engineering *SOS1* for the development of salinity-smart crops

Discovery of the dual role of *SOS1* and the salt concentration-dependent Na^+^ management strategies opened a new dimension of research on the engineering of salt-tolerant crops. In traditional approaches, salt tolerance generally focuses on overexpressing single transporters, often with tissue-specific effects (Pardo and Rubio, 2011). The recent breakthrough by Ramakrishna et al. (2025) suggests a promising strategy for the engineering of crops with enhanced *SOS1* trafficking to the EC and VC under salt stress. This strategy has potential if it can be achieved by fusing *SOS1* with trafficking signals or a system that regulates *SOS1* localization, as well as the engineering of crops that could maintain the proton gradient and regulate the *SOS1* activity. More specifically, the activity of *SOS1* in the EC could enhance the vacuolar Na^+^ sequestration efficiency.

## 6 Concluding remarks and future directions

This study explores new insights into the dual function of *SOS1* in the management of vacuolar Na^+^ in cells. The application of CryoNanoSIMS provides an opportunity to understand rapid response to salt stress and subcellular Na^+^ distribution, opening a new dimension of research on salinity stress. However, it is important to recognize the technical constraints of CryoNanoSIMS, including limitations in spatial resolution, quantification accuracy, and potential matrix effects, which should be carefully considered when interpreting the results. Insightfully, recognition of the dual function of *SOS1* opens a new avenue for the biotechnological application of *SOS1* in the development of salinity-smart crops. The path forward shows a clear understanding of cellular Na^+^ homeostasis linked to the recent finding of advance CryoNanoSIMS imaging, genetic, and engineering approaches for sustainable agricultural solutions. *SOS1*-mediated salt tolerance using molecular breeding or a genome-editing approach would be useful to plant breeders for sustainable agricultural crop production and for ensuring global food security. Despite recent progress in advanced imaging technology, there are several questions that need to be addressed for its further exploration. For instance, *SOS1* is crucial for *Arabidopsis* root meristem; simultaneously, other tonoplast-localized Na^+^/H^+^ antiporters such as *NHX1–4* are also expressed. Thus, there is a need to clarify whether *NHX1–4* could play a significant role in vacuolar Na^+^ management. Alteration in the cell switch could determine how rapidly cells switch between two different Na^+^ management strategies. Finally, time-course studies of salt stress and their temporal resolution could explore whether the alteration in vacuolar sequestration is immediate or opposite mechanisms are deployed. Further clarification of these queries will guide further direction and more in-depth understanding of salt stress research.
